# Enhancing communication skills for telehealth: development and implementation of a Teach-Back intervention for a national maternal and child health helpline in Australia

**DOI:** 10.1186/s12913-018-2956-6

**Published:** 2018-03-07

**Authors:** Suzanne Morony, Kristie Weir, Gregory Duncan, Janice Biggs, Don Nutbeam, Kirsten J. Mccaffery

**Affiliations:** 1 The University of Sydney, Sydney School of Public Health, Sydney, NSW Australia; 2 The University of Sydney, Wiser Healthcare, Sydney School of Public Health, Sydney, NSW Australia; 3 Eastern Health Clinical School, Medicine, Nursing and Health Sciences, Monash University, Box Hill, Melbourne, VIC Australia; 4 Healthdirect Australia, Sydney, NSW Australia; 5 The University of Sydney, Sydney Health Literacy Lab, Sydney School of Public Health, Sydney, NSW Australia

**Keywords:** Teach-back communication, Training, Self-reflection, Self-directed learning, Metacognition, Communication skills training, Professional development, Telenursing, Health-line, Teleadvice, Theoretical domains framework, TDF, Health literacy

## Abstract

**Background:**

Telehealth professionals require advanced communication skills, in part to compensate for lack of visual cues. Teach-Back is a best practice communication technique that has been recommended but not previously evaluated for consumer telehealth. We aimed to implement Teach-Back at a national maternal and child health telephone helpline. We describe the intervention and report telenurse experiences learning to use Teach-Back.

**Methods:**

We identified barriers (time, knowledge, skills, beliefs) and enablers (self-reflection) to using Teach-Back, and developed a novel training program to address these, guided by the Theoretical Domains Framework. We engaged maternal and child health telenurses to participate in a “communication skills” study. The intervention had two key components: guided self-reflection and a Teach-Back skills workshop. For the duration of the 7-week study nurses completed brief online surveys following each call, reflecting on both the effectiveness of their communication and perceived caller understanding. At the end of each shift they reflected on what worked well. Teach-Back knowledge, skills, and beliefs were addressed in a 2-h workshop using videos, discussion, and role play. We explored nurses’ experiences of the intervention in focus groups and interviews; and analysed transcripts and comments from the self-reflection surveys using the Framework method. This study forms part of a larger evaluation conducted in 2016.

**Results:**

In total 16 nurses participated: 15 were trained in Teach-Back, and 13 participated in focus groups or interviews. All engaged with both self-reflection and Teach-Back, although to differing extents. Those who reported acquiring Teach-Back skills easily limited themselves to one or two Teach-Back phrases. Nurses reported that actively self-reflecting (including on what they did well) was useful both for developing Teach-Back skills and analysing effectiveness of the techniques. Most wanted more opportunity to learn how their colleagues manage Teach-Back in different situations, and more visual reminders to use Teach-Back.

**Conclusions:**

Our theory-informed intervention successfully enabled nurses to use Teach-Back. Guided self-reflection is a low-resource method aligned with nurse professional identity that can facilitate Teach-Back skills learning, and could also be applied to other advanced communication skills for telehealth. Listening to multiple workplace-specific examples of Teach-Back is recommended for future training.

**Trial registration:**

ACTRN12616000623493 Registered 15 May 2016. Retrospectively registered.

**Electronic supplementary material:**

The online version of this article (10.1186/s12913-018-2956-6) contains supplementary material, which is available to authorized users.

## Background

The inability of patients to correctly recall information from a healthcare consultation is a well-known problem with significant consequences for treatment adherence. It is estimated that 40–80% of information is forgotten immediately or misremembered [1]. For example, while parents are typically able to remember their child’s diagnosis, their memory for a treatment protocol and actions to take remains poor [2]. These problems may be more pronounced for telephone consultations, given that up to 55% of the impact in a face to face consultation can be attributed to visual and non-verbal communication [3], and there is evidence that callers frequently misunderstand health information given by phone [4]. Telehealth consultations involve more frequent patient requests for information to be repeated [5]; however, people with low health literacy are typically less inclined to ask questions and may not indicate if they do not understand [6–8]. People experiencing acute stress or anxiety may also suffer temporary cognitive impairments that limit their ability to process information [1, 9, 10]. Telephone-based healthcare is expanding and can improve access, convenience and cost savings for both health care providers and clients [11, 12]; however, it also carries risks [13], and issues of patient safety in telehealth are largely unexplored [14]. The value of a telephone health service is questionable if callers immediately forget or misinterpret the information or instructions they have been given.

There is agreement that telehealth providers require high-level communication skills to compensate for lack of visual cues [13, 15]. There is currently no consensus on what their learning outcomes should be [16, 17], guidance on training methods is lacking [18, 19], and opportunities for further training remain limited [20]. One communication technique that has been recommended for consumer telephone consultations [3, 4] is Teach-Back, also known as the “interactive communication loop”. A key feature of Teach-Back is that the onus of ensuring information has been correctly understood rests with the health professional. Teach-Back requires information providers to invite consumers into the conversation, by asking individuals to explain back using their own words the information or action plans discussed. The healthcare provider can then add, clarify, or correct information as required. Teach-Back has been used effectively to improve accuracy of telephone communication between professionals [21] and to support child health outcomes in paediatric settings by improving parents’ comprehension of information [22]. It is regarded as an important patient safety practice [23], is widely recommended as a universal precautions approach in healthcare settings [24–29] and was recently ranked as the number one health literacy practice by experts [30]. We are not aware of previous efforts to train and evaluate the impact of Teach-Back in a consumer telehealth setting.

Previous research with healthcare providers has identified four key barriers to delivering Teach-Back: perceived time constraints [24, 31–34], quantity and complexity of information discussed [35], discomfort with phrasing [34, 36] (including consumer-reported discomfort [37]), and divided attention (of parents) [34, 35]. Enablers to the use of Teach-Back include a supportive work environment and practitioner self-reflection [38]. Critical self-reflection is an essential component of self-regulated learning. It is regularly recommended for nursing clinical skill development [39–41] and has been used in a number of nursing contexts (see [42] for a recent review) to bridge the gap between theory and practice [39]. Engaging nurses to purposively reflect on their practice while developing new communication skills may both acknowledge their professional competence [43] and encourage them to “test” the efficacy of the new techniques.

This paper describes the development and implementation of a theory-informed ‘Teach-Back skills’ program delivered to nurses operating a national maternal and child health telephone helpline. It is part of a larger study evaluating the effectiveness of Teach-Back for telehealth. We have previously reported nurse experiences using different Teach-Back phrases and with different kinds of calls at the helpline [44]; in this paper we report their experiences learning to use Teach-Back.

## Methods

### Setting

The setting was an Australian national telephone helpline operating from a single call centre. The Pregnancy Birth and Baby (PBB) telephone helpline offers free guidance and reassurance on pregnancy and parenting of children up to 5 years, and is delivered by Healthdirect Australia on behalf of the Australian Government. At the time of the study it was operated by the Royal District Nursing Service (RDNS), a state-funded community nursing organisation in Melbourne, Australia. Calls to the helpline include requests for information (e.g. foods or medicines to avoid during pregnancy), and self-management instructions (e.g. when to seek further assistance). Training opportunities were limited to a maximum of two hours face-to-face in small groups. This study was part of a broader health literacy initiative of Healthdirect Australia.

### Intervention development

We were guided by the Theoretical Domains Framework [45] and a model for developing a theory-informed implementation intervention [46], to examine: what (and whose) behaviours need to change; which barriers and enablers need to be addressed; which intervention components could respond to the barriers and enablers, and; how behaviour change can be measured and understood. This is detailed in Table 1 and summarised below.

**Table 1:** Description of barriers, enablers and intervention components for implementing Teach-Back with reference to the Theoretical Domains Framework

Using a theoretical framework, which barriers and enablers need to be addressed?	Within which theoretical domains do the barriers and enablers operate? (Domain numbering from [45])	Intervention components targeted at overcoming the modifiable barriers and enhancing the enablers
Barriers		
Lack of knowledge of how to use the Teach-Back method in this workplace setting	1. Knowledge 2. Skills 9. Goals 12. Social influences 14. Behavioural regulation	*Technique*: information provision, model/demonstrate the behaviour; rehearsal; encourage problem-solving *Mode*: Slides and workshop discussion; short (< 90 s) training video (× 2) *Content*: text and video examples of Teach-Back in practice including familiar scenario in this workplace. Role-play practice with Teach-Back. Facing away from each other to simulate telephone environment, swapping roles between caller/nurse, and changing partners.
Time constraints. Concerns about managing calls within time guidelines (i.e. 10 min)	6. Beliefs about consequences 11. Environmental context and resources	*Technique*: environmental modification, barrier identification, *Mode:* relaxing guidelines for maximum call duration for duration of study; workshop discussions *Content*: Call centre management advised temporary changes to call duration guidelines; workshop facilitators informed nurses of a previous study [36] that found Teach-Back often reduced consultation time (and why)
Difficulty phrasing. Nurse beliefs and attitudes about own communication effectiveness and asking for Teach-Back	2. Skills 3. Social/Professional role and identity 4. Beliefs about capabilities 6. Beliefs about consequences 7. Reinforcement	*Technique*: information provision, barrier identification *Mode*: facilitated workshop, guided discussion including discussing barriers to asking Teach-Back; offering a wide range of phrases to try *Content*: Acknowledging it can be difficult to ask for Teach-Back. Discussing that consumers typically do not mind or do not notice.
Volume and complexity of information discussed, caller needs (incl. Parents’ divided attention)	2. Skills 4. Beliefs about capabilities 6. Beliefs about consequences	*Technique:* information provision, encourage problem-solving *Mode:* illustrating situations where Teach-Back can be helpful; introducing “chunk and check” to break down complex information *Content*: video of distracted caller
Enablers		
Self-reflection	10. Memory, attention, and decision processes 11. Environmental context and resources 14. Behavioural regulation	*Technique:* encouraging self-reflection after each call *Mode:* online self-reflection surveys, email reminders to complete surveys; Teach-Back phrases on paper and in surveys *Content:* reflecting on efficacy of explanation and caller understanding (Additional file 1)
Supportive work environment	3. Social/Professional role and identity 4. Beliefs about capabilities 6. Beliefs about consequences 7. Reinforcement	*Technique*: linking rewards to the activities; encouraging nurses to experiment with different techniques; permission to fail *Mode*: self-reflection exercise linked to professional development rewards and professional identity as nurses; management support *Content:* performance is self-assessed with a focus on learning; call duration guidelines relaxed (see above)

The nurse behaviour we targeted for change was using the Teach-Back method with callers. To confirm that Teach-Back was not already being used at the helpline, JB and DZ audited a selection of helpline calls with reference to a checklist of Teach-Back behaviours and strategies prior to study commencement. To develop knowledge, skills, and beliefs with respect to why, when, and how to use Teach-Back, we conducted small-group workshops illustrating Teach-Back techniques, discussing barriers to using Teach-Back, and role-play practice (see below and Table 2). In order to make training cost-efficient we aimed to maximise nurses’ self-directed learning. We did this by encouraging nurses to self-reflect on their communication during each call, both before and after learning Teach Back, using an online survey to facilitate this (see below and Additional file 1).

**Table 2:** Overview of training materials and activities developed for the 2-h Teach-Back training session

Module 1 - Introduction to Teach-Back: Overview of evidence supporting Teach-Back, why asking “do you understand?” is ineffective to check understanding, emphasis on provider to explain properly, when to use Teach-Back, example phrases, and how to manage when someone cannot Teach-Back.
Module 2 – Teach-Back in practice: Why use Teach-Back over the phone (includes video described below), indications of caller engagement and understanding, role play activities, reflections on role play, strategies for mastering Teach-Back.
Video: (< 2 min) developed to illustrate Teach-Back in practice, with an articulate caller in a comfortable home being distracted by a young child. This was to highlight the importance of a universal precautions approach, because nurses cannot see what is going on behind the scenes or whether the caller is fully engaged, and ability to absorb information can be situational.
Handouts: Paper handouts were kept to a minimum for practical reasons. Nurses received 2 double-sided pages: one (blue) with a list of Teach-Back strategies and examples of what is Teach-Back and what is not; the other (orange) with suggestions for when to use Teach-Back and what to do when the caller cannot teach back.
Role play: Nurses had their backs to each other during the role play to simulate the telephone environment. One person was the “caller”, and used a familiar scenario. The other person was the “nurse” and used the Teach-Back method to confirm understanding. Following the role-plays nurses discussed the experiences as a group. Group 1 had a single role-play session. Group 2 had two role-plays, and participants chose different partners for the different role plays.

### Teach-back workshop

Training in Teach-Back theory and skills was a single 2-h group workshop conducted by 2 facilitators (SM, a research psychologist experienced with call centre operations, and GD, a research pharmacist experienced in Teach-Back training). Training groups (7–8 nurses) were held two weeks apart, and used a collaborative (rather than didactic) approach. Workshop materials were evidence-based Teach-Back training materials developed elsewhere [36] and adapted for the helpline nurses, and consisted of theory, discussion, and practice (Table 2), including barriers to Teach-Back identified from previous research (Table 1). A short video was developed illustrating Teach-Back in a familiar scenario (a caller to the helpline being distracted by her child). Suggested Teach-Back strategies were adapted from an online resource [38]. Group facilitators invited nurses to identify call scenarios that might be suitable for Teach-Back and discover ways of asking for Teach-Back that felt natural. Training discussions acknowledged that not all calls would be amenable to Teach-Back and nurses should use their professional judgement.

### Self-reflection surveys

The online self-reflection survey was introduced in a brief video distributed by email, and nurses were advised self-reflection activities could contribute towards their annual continuing professional development (CPD) hours. For the duration of the 7-week study nurses were encouraged to reflect after each call on both the quality of their explanation and their perception of caller comprehension (Additional file 1). Following the Teach-Back workshop (2–4 weeks into the study), additional questions asked which Teach-Back strategies were (and were not) used and why. The surveys also acted as a reminder to use Teach-Back. At the end of each shift, open questions invited nurse comments about their experiences and any communication strategies that worked well (or not) for them. Emails from the research team encouraged participating nurses to complete self-reflections and report any Teach-Back phrases they found helpful. One such technique, *“So just to recap, what will you do now? And when do you think you need to take further action?*” was added to the online list of Teach-Back strategies at the start of week 4 (one week after the first group had been trained).

### Participants

All nurses normally working at least 3 h per week at the helpline (*n* = 18) were invited to participate. Three of these nurses, who worked infrequent shifts or had leave planned during the study period, declined participation. Written informed consent was obtained for all participants, and ethical approval was obtained from the Royal District Nursing Service (150013) and The University of Sydney (2016/083). Consenting nurses were informed via a short online video that this was a research study to test effectiveness of training in new communication skills, and that they had been randomly allocated to one of two groups for training purposes. Group 1 nurses were asked to avoid sharing workshop materials and techniques for the two weeks before the second group was trained, to prevent contamination.

### Data collection

Self-reflection data was collected in real time using an online survey programmed in Qualtrics survey software (Qualtrics, Provo, UT, ©2016) (Additional file 1). Following the trial conclusion we conducted focus groups (onsite, 2 h duration) and interviews (telephone, 15–20 min), with participating nurses and their team leader using a semi-structured topic guide, exploring experiences of the training and of using Teach-Back (Additional file 2). The two focus groups (3–5 nurses per group) were held on the final day of the trial, and nurse attendance was paid. Telephone interviews with nurses unable to attend focus groups (*n* = 4) and the team leader (*n* = 1) were conducted by KW (f), a public health researcher, during the following weeks. Field notes were taken (by SM) during focus groups, and both focus groups and interviews were audio recorded and transcribed. The study used a phenomenological methodology.

### Analysis

Transcripts of the interviews and focus groups were analysed using a Framework approach [47], a matrix based method of thematic analysis, with participants as rows and themes as columns. Firstly, one researcher (SM) summarised (and verified with GD) field notes taken during the focus groups (familiarisation) and developed an initial coding framework (identification) guided by the research questions. SM applied an iterative process of coding data using NVivo11 and adding to the coding framework, identifying themes (indexing), then summarising in a thematic matrix (charting). When all of the data was coded, the framework was examined within and across themes and participants by SM and KW (mapping and interpretation) using inductive and deductive methods. Rigour was addressed throughout this process by ensuring a detailed documentation of the analysis process, constant comparison of data and continuous discussion of emerging and final themes. Relevant open-response data collected from the self-reflection surveys was added to the Framework. The Consolidated Criteria for Reporting Qualitative Research (COREQ) [48] were used to ensure quality of the research process. Quantitative data from the self-reflection surveys was examined using descriptive statistics.

## Results

### Participant characteristics

Training participants were 16 female maternal and child health nurses, including the team leader who oversaw operations. Focus group (*n* = 8) and interview (*n* = 5) participants were 13 nurses (including the team leader) aged 37–61 years (mean 55.1) who had been registered as nurses for 13–41 years (mean 29.5 years) and working in maternal and child health for between 7 months and 33 years (mean 14.2 years). Two had worked on a helpline previously. Number of shifts nurses worked per week ranged from 1 (3–4 h) to full-time (team leader); training groups were balanced by average hours worked per week. Two nurses who attended training declined to be interviewed, saying they had not had much opportunity to use Teach-Back due to rostering and leave. One was on leave and could not be contacted.

All nurses made an effort to engage with the online surveys. Responses were inconsistent however, ranging from 14 to 608 call reflections per nurse as captured in the online survey (0 to 265 reflections following Teach-Back training) and 1 to 20 end-of-shift reflections per nurse. This reflected differences not only in hours worked during the study period but also in interpretation of and engagement with the self-reflection activities. We examined distributions of the quantitative data from the self-reflection surveys (Additional file 1) to identify how frequently nurses reported using Teach-Back (following training). Most nurses (*n* = 10) most commonly responded that they used Teach-Back “fully”; for two others the mode was “yes-somewhat or partially”. This suggests that overall nurses made considerable effort to use Teach-Back strategies. Two nurses most frequently responded that they did not use Teach-Back because they felt it was “inappropriate” for that call.

We explored nurse experiences learning the new skills, what worked, and what could be improved for learning to use Teach-Back (Additional file 2). We identified three key themes in the data: learning to use Teach-Back, using self-reflection for skill development; and suggestions for future. In the nurse quotes reported below, we denote data from focus groups and interviews “N”, and comments from the self-reflection surveys “SR”.

### Changing practice: Learning to use teach-back

Confirming a lack of prior knowledge of Teach-Back, all but one nurse reported she had not learned or used Teach-Back previously.

“*It was all teach, not teach-*back”. (N5).

Prior to the Teach-Back workshop nurses’ self-reflections described using communication techniques such as active listening, confirming understanding, and asking for clarification; but some noted they were not always sure if clients understood or will follow through. Following training, nurses reported asking callers what they will do following the call, and indicated they found added value from Teach-Back techniques.

*“Clients showed me that they understood my instructions. It helps me feel more confident that the information I am conveying is coming across accurately.” (SR).*

Several nurses reported speaking to a client who was unable to Teach-Back the information. Nurses commented both on the perceived impact on the client “*I think she felt embarrassed and felt like she had been put on the spot*” (SR), and expressed interest or surprise that their message had not been received “*oh my goodness she could not recall one word I said!” (SR).*

A few nurses reported they were comfortable using Teach-Back within one or two shifts, but many struggled to find wording that sounded or felt natural to them, or felt that they were imposing Teach-Back on callers. One nurse reported feeling overwhelmed by numerous possible Teach-Back strategies, saying this interfered with her ability to focus on the call and implement Teach-Back. One technique reported to be effective was to read through the list of suggested phrases, identify a couple that sounded “like me”, and restrict Teach-Back to those phrases.

*“Well try not to get too bogged down in how to go about it, find something that's natural and easy that will just sort of roll off the tongue. It's going to take time, isn't it …” (N2).*

Nurses commented that they enjoyed the approach to training and this was a factor in them embracing it. They made frequent reference to the training video because it highlighted the contextual factors of the unseen caller that may interfere with information transfer. The majority thought the social environment of face to face sessions were important for introducing a new skill such as Teach-Back. One nurse commented that the training had too much theory and not enough role playing. Nurses frequently mentioned that they enjoyed the practical application of skill during role play, because “*nurses are such practical people”. (N3).*

“*Yeah, two times was good I think…and the fact that we swapped partners as well, so you didn't just do it with the same person, that seemed to work well.…”(N6).*

Comfort with using Teach-Back did not appear to be related to prior experience or the overall number of shifts worked. In the self-reflections nurses commented they were “getting better” or “more comfortable” with using the techniques over time, indicating practice may help overcome some of these barriers. When Teach-Back was not considered appropriate nurses indicated this was because of a distressed child that needed attention, because the caller taught back spontaneously, or the call was a transfer to another service. At the end of the shift some nurses reflected they did not use Teach-Back as often as they thought they “should”.

### Self-reflection to support skill development

One nurse described the self-reflection that is part of nurse training:

*“We are taught to do self-reflection as nurses.. [if].. there was some issue you need to unpack it yourself or improve what actually happened in that call...” (N9).*

In focus groups and interviews nurses commented that the self-reflection surveys were helpful initially but became boring and repetitive after a while. Despite this, many nurses commented that this was the first time they had truly reflected on their own calls. One nurse reported she enjoyed the opportunity to reflect on “good” calls, and others described the self-reflections as therapeutic by helping remove any worries about how clients were going to manage the situation or what they would do after hanging up.

*“And it produces closure to the day, to the shift, [so you don’t] go home worrying that somebody’s going to do something at three o’clock in the morning…* ”*(N3).*

Some mentioned it was difficult to keep up with self-reflections when multiple calls were coming through in rapid succession. The multiple tasks sometimes resulted in them forgetting to use Teach-Back, self-reflect, and/or recruit the caller for follow-up. Some nurses commented that they tended to self-reflect instinctively, but others mentioned it was helpful to have this focus and think about their own satisfaction with the call.

“*I think the reflecting was good to start with, I don’t know that we need to do it anymore, but I wonder if you need to revisit it…...every couple of months or twice a year or once a year.”(N6).*

### Suggested improvements

Nurses mentioned that visual reminders such as posters or a field in their computer system that they need to check each time would be helpful to maintain Teach-Back practice. Several mentioned it would be helpful to have a follow-up session with the trainers to monitor progress and to learn from their colleagues. Some thought Teach-Back could be incorporated into their performance management and professional development, or could be part of the initial training they underwent when starting work at the helpline. A few thought training could be conducted via video or podcast but found added value with the group face to face interaction. All nurses commented that learning from colleagues and listening to real examples was very helpful for learning how to apply new communication techniques in different contexts.

*“I find just the listening [to other nurses] works really well….because I think you just learn to do it on the run.” (N2).*

Although there was widespread agreement that they learned the most from each other, nurses noted opportunities to do this were limited because they were rarely together in the same room. Some suggested that the team leader, who routinely reviews calls for quality audits, could identify call recordings in which Teach-Back was used very well (or very poorly) for training purposes; others thought there may be also value in listening to recordings of their own calls:

*“Even the times where I thought, I didn't say that correctly, they gave me the responses that I expected and I thought obviously I must have sounded better than what I thought. So if you can reflect on that and listen to yourself, that's a good teaching tool.” (N1).*

### Integration with theoretical domains framework

In Table 1 we presented the barriers and enablers to using Teach-Back identified in the literature, and strategies to manage them informed by the revised Theoretical Domains Framework (TDF). Here we discuss the nurse data with reference to the TDF (domains denoted “x)” corresponding to numbering in [45]). With respect to nurse behaviour change, the primary objectives in this study were to increase nurses’ 1) knowledge, 2) skills, and 4) beliefs about capability to use Teach-Back in telephone consultations. In order to embed Teach-Back into professional practice it primarily targeted 14) behavioural regulation (self-monitoring), 3) professional role and identity, and 6) beliefs about consequences. The option to count self-reflection activities towards continuing professional development accreditation also acted as a reward, i.e. 7) reinforcement.

Feedback from nurses suggests the program was largely successful in developing 1) knowledge and 6) beliefs about consequences of Teach-Back; however, nurses reported varying levels of 2) skill, 13) comfort and 4) beliefs about capabilities for using Teach-Back. The wide range of Teach-Back phrases offered as a resource for nurses negatively impacted 10) attention and memory processes for some, a difficulty that may have been compounded by the additional requirement to complete self-reflection surveys. Explicit instructions to focus on only one or two Teach-Back phrases may overcome this. Future implementation may add salient 11) environmental cues such as visual reminders in the workplace like posters or a “did you use Teach-Back” checkbox in the customer relationship management (CRM) software. Future iterations should also facilitate opportunity to learn from colleagues for both 2) skills and 12) social influences. This might include formal training with colleagues by telephone, for example telephone role-play scenarios - although the social impact of a telephone conversation is likely different from a group session.

## Discussion

Our findings indicate that our theory-informed intervention combining ongoing deliberate self-reflection with a single 2-h workshop was largely successful for experienced maternal and child health nurses to develop confidence and skills to use Teach-Back in a telephone helpline for the first time. Nurses perceived they were more effective at ensuring comprehension when using Teach-Back, which may have been enhanced by their deliberative focus on clear explanation and caller understanding with the self-reflection surveys. Nurses reported that the training workshop and self-reflection surveys were helpful for developing Teach-Back skills, and were unanimous in wanting more opportunities to learn from peers on how to use Teach-Back in a range of situations relevant to their specific work environment. This was particularly true for nurses who struggled to use Teach-Back in a way that sounded natural to them. Nurses who reported success with finding their “voice” generally focused on one or two phrases they were comfortable with.

Although the usual target of reflective practice is clinical judgment, this study provides qualitative evidence that a simple online program encouraging self-reflection has value for enhancing communication skill development. This is aligned with current thinking on moving away from a “training mindset” in communication skills development and focusing instead on building on learners’ pre-existing knowledge and skills through “reflection, experiential work, and self-directed learning” [43]. We are not aware of any other studies prompting real-time self-assessments for communication skills training or telenursing (although a variation of this technique has previously been used in undergraduate statistics teaching [49]). Most nurses had not engaged in this kind of deliberate self-reflection for their role in delivering telephone advice to consumers, or reflection on what went well. Communication skills training for medical professionals has elsewhere been shown to increase communication self-efficacy [50]. Reflecting only on negative experiences can promote loss of confidence [39], but reflecting on positive experiences may balance this. In this study nurses reported benefits from reflecting on what they did well. The surveys were considered annoying as well as useful, so any future implementation will need to find an appropriate balance. The focus groups were another opportunity for reflection, and nurses appeared to enjoy learning from and with each other, which may have helped consolidate learning.

The organisational context was an important factor in changing behaviour, including implementation momentum from key stakeholders. To reduce time pressure on nurses the management team temporarily relaxed guidelines and consequences for call duration (although nurses still found this a challenge as reported elsewhere [44]). External reinforcement, such as feedback by colleagues or supervisors was not built into this training, and nurses relied on self-reflections and reactions from their clients to monitor their success using Teach-Back. This is consistent with a paradigm of nurse education that harnesses reflective practice to equip learners to develop their own expertise [51].

### Limitations

All data was self-report, which carries an inherent risk of bias. Participants in this study were experienced nurses who were able to engage with self-regulated learning in a workplace supportive of research and innovation. It may be less effective or require more structure for other learners, or in work environments that do not support professional development and standards [39]. Finally, nurses may have been more motivated to participate because this was a research study. A key feature of the self-reflection data collected in this study is that it went directly to the research team and was only reported in summary form, so there was no possibility of evaluation by managers or colleagues. Fear of judgment is one obstacle to encouraging self-reflections [39] and an in-house self-reflection survey may have been less effective. Conversely, it could alert team leaders to staff who may need more help with training. Formative assessment for self-regulated learning typically involves feedback, and this study did not objectively evaluate the calls or deliver any feedback on reflections.

## Conclusions

With the increased use of telehealth services it is critical to continue to develop strategies to improve the safety and quality of telephone health advice and embed them into routine clinical governance [52]. In this study, telehealth nurses were able to build the Teach-Back method into their practice, following a theory-informed intervention consisting of a brief training workshop combined with ongoing self-reflection. This intervention may also be adapted for other advanced communication skills and in other telehealth settings.

## Additional files

Additional file 1: Items from online self-reflection survey completed following each call and each shift. (DOCX 15 kb)

Additional file 2: Complete interview guide for nurse focus groups and telephone interviews. (DOCX 16 kb)

## Abbreviations

COREQ: Consolidated Criteria for Reporting Qualitative Research
CPD: Continuing Professional Development
CRM: Customer Relationship Management
PBB: Pregnancy Birth and Baby
RDNS: Royal District Nursing Service
TDF: Theoretical Domains Framework
