# Supplementary material for: Enhancing communication skills for telehealth: development and implementation of a Teach-Back intervention for a national maternal and child health helpline in Australia
Source: BMC Health Serv Res. 2018 Mar 7;18:162. doi: 10.1186/s12913-018-2956-6 (PMC5842621; doi:10.1186/s12913-018-2956-6)
Supplement: Supplementary file 1 — Items from online self-reflection survey completed following each call and each shift. (DOCX 15 kb) [file 12913_2018_2956_MOESM1_ESM.docx]

Additional file 1: Self-reflection survey questions

| **Reflections after each call**  *Before Teach-Back workshop: two questions following each call (5 response options)*   1. How effective do you think you were at communicating the information? 2. How well do you think the caller understood your instructions or recommendations?    1. Extremely    2. Quite a bit    3. Somewhat    4. A little bit    5. Not at all   *After Teach-Back workshop: two additional questions (multiple responses accepted for question 4)*   1. Did you use Teach-Back?    1. Yes - fully    2. Yes – somewhat/partially    3. No – forgot    4. No – inappropriate    5. No – too difficult    6. No – other (describe) 2. Please indicate if you used the following Teach-Back strategies:    1. I want to be sure I explained everything clearly. Can you explain it back to me so I can be sure I did?    2. How would you explain this to another parent with the same question?    3. What will you tell your partner about our conversation today?    4. We’ve gone over a lot of information. In your own words, please review with me what we talked about    5. What questions do you have?    6. What are you going to do… when you get off the phone/tomorrow/next feed, sleep, etc?    7. Other (please specify)   **End of shift reflections**  Nurses were asked to reflect on their experiences during the shift. They could also respond to these questions at any time during their shift:  *Before Teach-Back workshop*   1. How was your experience communicating information to your callers today? Tell us about any positive or negative experiences? 2. Are there any techniques you used today that you thought worked particularly well (or poorly)? Please tell us about them   *After Teach-Back workshop*   1. What was your experience of using Teach-Back today? Tell us any experiences (positive or negative)? What would you do differently? 2. Are there any Teach-Back techniques that work well for you that you would like us to share with other staff? Please describe. |
| --- |
