# Supplementary material for: Enhancing communication skills for telehealth: development and implementation of a Teach-Back intervention for a national maternal and child health helpline in Australia
Source: BMC Health Serv Res. 2018 Mar 7;18:162. doi: 10.1186/s12913-018-2956-6 (PMC5842621; doi:10.1186/s12913-018-2956-6)
Supplement: Supplementary file 2 — Complete interview guide for nurse focus groups and telephone interviews. (DOCX 16 kb) [file 12913_2018_2956_MOESM2_ESM.docx]

Additional file 2: Interview guide for nurse focus groups and telephone interviews*

**Teach-Back**

1. How did you find the experience of using Teach-Back in this trial?
2. What are the benefits or disadvantages of Teach-Back?
3. How long did it take to get comfortable using it?
4. Is Teach-Back something you would like to work with in future?
5. Any thoughts on a future rollout to other Healthdirect (or other telephone) services?
6. How well do you think Teach-Back is suited to this (contact centre) environment?

Barriers & Facilitators

1. Did you notice a difference in the way clients responded to the information you delivered?
   - How were calls different during Teach-Back?
2. Describe any Teach-Back strategies that worked particularly well (or poorly) for you?
3. What type of calls do you think suited Teach-Back?
   - When was Teach-Back appropriate/not appropriate?
4. How do you manage shorter calls?
   - Is Teach-Back suitable? What else (e.g. a different communication technique) is needed to close off the call?
5. What difficulties did you encounter when delivering Teach-Back? How did you resolve them?
6. Do you think asking for Teach-Back added time to your calls?
7. Did any callers mind/were awkward when you used Teach-Back? How did you handle this?

Training feedback

1. What was your experience of the self-reflective exercises? Was it useful? Annoying? Will you continue to self-reflect following the study period?
2. What was your experience of the training? Are there any changes you would suggest?
   - Could any be done online?
3. Can you suggest any additional support/resources that would help the ongoing use of Teach-Back?
4. Do you have anything important you would like to tell us?

Other

1. Did you talk with any of the other nurses about the study?
   - Were they in same or different group?
2. Did you share Teach-Back techniques? (after training was complete)
3. What was your experience of working during the crossover period? (i.e. middle 2 weeks of the study when half the nurses were doing Teach-Back and half not).
4. What questions do you have about the study?

* Nurse (and caller) experiences with different Teach-Back phrases and for different kinds of calls are reported in: Morony, S., Weir, K., Duncan, G., Biggs, J., Nutbeam, D., & McCaffery, K. (2017). Experiences of Teach-Back in a Telephone Health Service. *HLRP: Health Literacy Research and Practice*, 1(4), e173-e181. DOI 10.3928/24748307-20170724-01
